# Prevalence of sleep bruxism in children: A systematic review

**DOI:** 10.1590/2176-9451.19.6.054-061.oar

**Published:** 2014

**Authors:** Eduardo Machado, Cibele Dal-Fabbro, Paulo Afonso Cunali, Osvaldo Bazzan Kaizer

**Affiliations:** 1 Federal University of Santa Maria, Masters student in Dental Sciences/Prothesis, Federal University of Santa Maria (UFSM); 2 Federal University of São Paulo, PhD in Sciences, Federal University of São Paulo (UNIFESP); 3 Federal University of Paraná, Professor, Undergraduate and Postgraduate program, Federal University of Paraná (UFPR). Coordinator, Postgraduate program in TMD and Orofacial Pain, UFPR; 4 UFSM, Professor, Undergraduate and Postgraduate program, UFSM

**Keywords:** Sleep bruxism, Bruxism, Prevalence, Child

## Abstract

**INTRODUCTION:**

Prevalence of sleep bruxism (SB) in children is subject to discussions in the literature.

**OBJECTIVE:**

This study is a systematic literature review aiming to critically assess the prevalence of SB in children.

**METHODS:**

Survey using the following research databases: MEDLINE, Cochrane, EMBASE, PubMed, Lilacs and BBO, from January 2000 to February 2013, focusing on studies specifically assessing the prevalence of SB in children.

**RESULTS:**

After applying the inclusion criteria, four studies were retrieved. Among the selected articles, the prevalence rates of SB ranged from 5.9% to 49.6%, and these variations showed possible associations with the diagnostic criteria used for SB.

**CONCLUSION:**

There is a small number of studies with the primary objective of assessing SB in children. Additionally, there was a wide variation in the prevalence of SB in children. Thus, further, evidence-based studies with standardized and validated diagnostic criteria are necessary to assess the prevalence of SB in children more accurately.

## Abstract (pt)

**INTRODUÇÃO:**

a prevalência de bruxismo do sono (BS) na população infantil é tema de discussões na literatura.

**OBJETIVO:**

assim, este trabalho, por meio de uma revisão sistemática, teve como objetivo avaliar, de forma crítica, a prevalência de BS em crianças.

**MÉTODOS:**

foi feito um levantamento nas bases de pesquisa MEDLINE, Cochrane, Embase, PubMed, Lilacs e BBO, entre os anos de 2000 e fevereiro de 2013, com enfoque em estudos que avaliaram especificamente a prevalência de BS em crianças.

**RESULTADOS:**

após a aplicação dos critérios de inclusão, chegou-se a quatro estudos. Entre os artigos selecionados, as taxas de prevalência de BS variaram de 5,9% a 49,6%, sendo que essas variações apresentaram possíveis associações com os critérios diagnósticos para BS.

**CONCLUSÃO:**

verifica-se um número reduzido de estudos que apresentaram o objetivo primário de avaliar o BS em crianças, além de uma grande variação nas taxas de prevalência. Torna-se necessária a realização de novos estudos, baseados em métodos de evidências e com critérios diagnósticos padronizados e validados, para avaliar com maior precisão a prevalência do BS em crianças.

## INTRODUCTION

Sleep bruxism (SB) is classified as a movement disorder related to sleep.^1^ This parafunction is characterized by non-functional teeth contact, manifesting by grinding or clenching of teeth. It is not a disease, but when exacerbated may lead to an imbalance of the stomatognathic system. Several therapeutic modalities have been suggested, but there is no consensus about the most efficient.^2^

The pathophysiology of SB is still unknown. It is considered multifactorial with potential influences of the central nervous system (CNS), including oral motor activities, regulation of sleep-wake cycle, autonomic and catecholaminergic as well as genetic and psychosocial influences. The role of dental occlusion remains controversial. The presence of EEG and cardiac autonomic activations suggests that SB is a consequence of micro-arousals.^3^

Polysomnographic findings of patients with SB include rhythmic or tonic activity of the masseter and temporal muscles during sleep and may occur at any stage, being more common in stages 1 and 2 of the non-REM or NREM (non-rapid eye movements) sleep. Sleep architecture is usually normal, but many times there is an increase in micro-arousals, number of changes in sleep stages and heart rate.^3^^,^ 4

Sleep bruxism is subject to constant discussion not only among dentists, but also in other health areas due to potential etiologic associations. Epidemiological studies with different methodologies and populations have been conducted, for this reason, the prevalence of SB varies in different age groups. In young adults aged between 18 and 29 years old, it is of 13%, reducing to 3% in individuals over 60 years of age.^5^ Still, when sleep bruxism is related to children, major doubts remain. Due to variations in the prevalence of bruxism in children, a systematic and critical analysis of current literature is necessary to obtain more accurate data. Thus, the aim of this systematic review is to discuss, based on scientific evidence, the real prevalence of sleep bruxism in children.

## MATERIAL AND METHODS

A computerized search was conducted in MEDLINE, Cochrane, EMBASE, Pubmed, Lilacs and BBO from January 2000 to February 2013. The research descriptors used were: "sleep", "bruxism", "child" and "prevalence", all of which were crossed in search engines using the boolean operators AND, OR or NOT. The initial list of articles, assessed by title and abstract, was submitted for review by two independent reviewers who applied inclusion criteria to determine the final sample. Should there be disagreement between the results of reviewers, a third reviewer would be required to read the full version of the article.

When selecting the sample, the following inclusion criteria were applied:

" Studies with the primary objective of assessing the prevalence of sleep bruxism in children.
" Individuals aged between 0 and 12 years considered as children.
" Studies using any of the following SB diagnostic criteria: history, questionnaire or interview with parents, clinical assessment or polysomnography.
" Studies published between January 2000 and February 2013 without language restrictions. The period was chosen due to an attempt to retrieve studies with more precise and accurate methodological criteria and new discoveries about SB over the past few years.
" In case of multiple publications originating from the same study, only the main and most specific publication was considered.

The following exclusion criteria were also applied:

" Epidemiological studies aiming to assess the prevalence of other sleep disorders, oral habits, occlusal factors and temporomandibular disorders (TMD) in conjunction with the assessment for SB.
" Studies with the primary objective of assessing sleep bruxism in children with congenital and chromosomal syndromes, permanent systemic changes, cerebral palsy and psychiatric disorders.

## RESULTS

**Figure 1.**
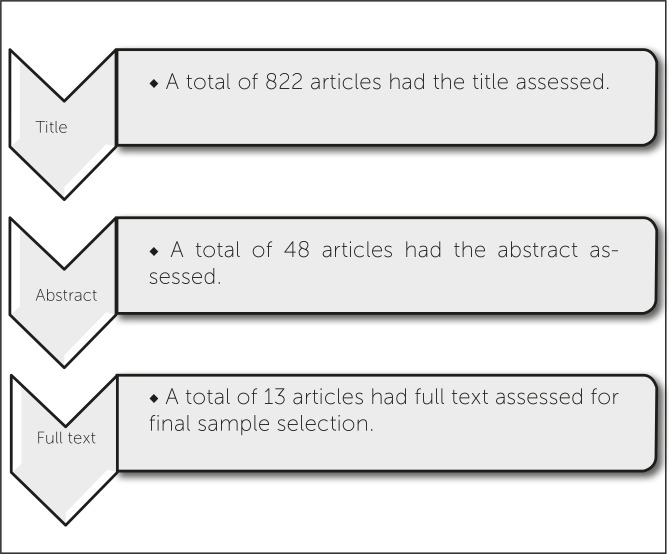
Flowchart of initial search.

After applying the inclusion criteria, the final sample comprised four studies. Kappa index of agreement between the authors was 1.00, without the need for evaluation by a third reviewer. The flowchart of the initial search can be seen in Figure 1. First, articles were assessed by title and abstract. Articles that did not meet the inclusion criteria for the systematic review were excluded. The main reason is that some articles did not have the prevalence of SB as a primary objective, but focused on SB in association with other conditions. After the first two selection processes, the studies were analyzed by a reviewer who read the full version of the article. Once again, articles that did not have the prevalence of SB as the primary objective of the study were excluded.

Characteristics and results of the studies are shown in Tables 1 and 2.

**Table 1.** Characteristics of studies included in the final sample.

Study	Sample size	Study location	Sample characteristics	SB diagnosis criteria
Fonseca et al,^6^ 2010	170 children attending municipal kindergartens	Study conducted in the rural area of Itanhandu, SP, Brazil	Mean age of 4.37 ± 1.69 years (51.76% girls)	Clinical examination according to the American Academy of Sleep Medicine associated with a questionnaire filled in by parents
Serra-Negra et al,^7^ 2010	652 children randomly selected from public and private schools	Study conducted in Belo Horizonte, MG, Brazil	Children aged between 7 and 10 years (52% girls)	Parents’s report based on a questionnaire according to the criteria of the American Academy of Sleep Disorders
Lam et al,^8^ 2011	6389 questionnaires filled in by patient’s parents	Study conducted in the districts of Shatin and Tai Po in Hong Kong, China	Mean age of 9.2 ± 1.8 years (50.6% boys)	Parents’ validated questionnaire (HK-CSQ)
Insana et al,^9^ 2013	1953 preschool children and 2888 first graders, and a sub-sample of preschool children (n = 249)	Study conducted in Jefferson County, Kentucky, USA	Preschool children (aged between 2.5 - 6.9 years); first graders (aged between 3 - 8.6 years); sub-sample of preschool children (aged between 2.87 - 6.11 years )	Parents’ questionnaire and additional behavioral and cognitive assessments in the sub-sample of 249 preschool children

**Table 2.** Results of the studies included.

Study	SB prevalence	Important findings	Study limitations	Study suggestions
Fonseca et al,^6^ 2010	15.29% (n = 26) were diagnosed as bruxists	Positive correlation was found between restless behavior and the presence of SB	Tooth wear may not reveal the actual level of SB. The study did not perform polysomnography evaluation. Memory biases	Association between clinical examination and parents’ questionnaire for SB diagnosis
Serra-Negra et al,^7^ 2010	Bruxism was prevalent in 35.3% (n = 230)	More than half of children without SB (55.2%) were of low socioeconomic background	The study did not perform clinical or polysomnography evaluations in children. Memory biases	The high prevalence of 35.3% reveals the need for further research on the subject
Lam et al,^8^ 2011	SB ≥ 3 episodes per week, showed a prevalence of 5.9% in children from Hong Kong	SB was more prevalent among boys and decreased with age. It was associated with several medical conditions, neuropsychiatric sequelae and sleep disorders	The study did not perform polysomnography evaluation. Memory biases	Further prospective studies are needed to assess the association between SB and other medical conditions
Insana et al,^9^ 2013	36.8% of preschool children and 49.6% of first graders reported episodes of bruxism at least once a week	Pediatric sleep bruxism may function as a warning sign for potential adverse health conditions	The study did not perform polysomnography evaluation. Memory biases	Future research may benefit from objective measurement of SB

Fonseca et al^6^ conducted a cross-sectional study with 170 children and a statistical power of 91.42%. This population of 170 children had a mean age of 4.37 ± 1.69 years, of which 88 (51.76 %) were girls. A total of 15.29% (n = 26) were considered bruxists as a result of this study: 15 boys (57.69%) and 11 girls. The average duration of breast feeding was 4.4 ± 0.25 months. Only 10% of the study population was on medication and 46.47 % exhibited restless behavior. The behavior of children was assessed by a questionnaire applied to children's parents. SB and behavior were positively correlated (P <0.001), as 73.1 % of bruxists exhibited restless behavior. Patients' sex (p = 0.595) did not correlate with SB. There was no correlation between children's behavior and medication (p = 0.573) or between SB and medication (p = 0.573). There was no correlation between the duration of breast feeding and restless behavior (p = 0.102), SB (p = 0.565) or medication (p = 0.794).

Serra-Negra et al^7^ also conducted a cross-sectional study with a sample of 652 children aged between 7 and 10 years old in both public and private schools of Belo Horizonte - Brazil. SB in children was reported by parents based on the criteria of the American Academy of Sleep Disorders. The Social Vulnerability Index, obtained by municipal databases, was used for social classification of families. SB was diagnosed in 230 children, with a prevalence of 35.3%. Among the 652 children, 340 (52%) were girls and 312 (48%) boys, predominantly of 8 years of age (84.2%). SB was diagnosed in 56.5% of girls and 43.5% boys. Most families were of low social vulnerability (54.2%), while 45.8% were of high social vulnerability. More than half of children without SB (55.2%) were of low socioeconomic background.

In the study by Lam et al,^8^ the authors selected a representative sample with socioeconomic background similar to the rest of Hong Kong. Children's parents were asked to complete the *Hong Kong children sleep questionnaire* (HK-CSQ), a validated sleep questionnaire that includes demographic and socioeconomic data, frequency of sleep disorders in the last year and the parents' opinion on whether children were hyperactive or bad-tempered, as well as children's academic performance. Regarding the socioeconomic level, including parental education, occupational status, marital status and residential environment, there were no differences between SB and non-bruxists (P >0.05). Neurobehavioral characteristics, including hyperactivity (adjusted for age and sex OR [95% CI] = 1.61 [1.25 - 2.07]), bad temper (adjusted OR [95% CI] = 1.69 [1.35 - 2.12]) and poor academic performance (OR adjusted [95% CI] = 1.22 [1.03 - 1.43]) were more common in patients with SB. They were also more likely to have chronic diseases, allergic rhinitis, asthma and upper respiratory tract infections (P < 0.05).

Insana et al^9^ assessed a convenience sample of which participants were recruited from two populations in Jefferson County, Kentucky / USA. One population comprised preschool children (n = 1953, M = 4.3 ± 6 [range: 2.5 - 6.9] years) while the other population attended first grade classes in public schools (n = 2888, M = 6.2 ± 0.5 [range: 3.0 - 8.6] years). All guardians answered a questionnaire about children's sleep and health. Data from a subgroup of children at preschool age (n = 249, M = 4.5 ± 0.7 [range: 2.87 - 6.11] years) were also examined. The parents of these children completed a report on the behavior of their child (Child Behavior Checklist - CBCL), whereas children completed neurocognitive assessments (Differential Ability Scales - DAS). Overall, 36.8% of preschool children were reported as bruxists at least one night a week, and 6.7% were reported as bruxists for more than four nights a week. Conversely, 49.6% of first-graders were reported to have SB at least one night per week, and 10.7% were reported for more than four nights a week. As for pre-school children, internalizing behaviors (i.e., anxiety, depression, withdrawals and somatic complaints) were independently associated with SB. Sleep bruxism was associated with health problems and health problems were associated with neurocognitive performance. The Sobel test for mediation did not identify a significant indirect relationship between SB and neurocognitive performance (Sobel = -1.49, P = 0.14).

## DISCUSSION

Dentistry has been increasingly inserted into a context based on scientific evidence. Thus, studies should use methodological criteria that qualify the evidence, including tools such as randomization, sample size calculation, calibration, blinding and control of involved factors.^10^ In addition, epidemiological studies on sleep bruxism should use standardized and validated diagnostic criteria. All information about the methods and diagnostic criteria adopted by authors should be available to the reader's appreciation.

Diagnosis of SB is primarily achieved by patient's history and physical examination. It might be complemented by polysomnography. Patient's history should include the study of sounds produced as a result of grinding or clenching, as reported by the patient's partner or guardian; morning facial pain or discomfort; headache; teeth sensitivity to hot or cold food; and the presence of fracture or dental restoration. Tooth wear, gingival recession, masticatory muscles hypertrophy and presence of joint sounds in TMJ palpation may be present on physical examination, especially in more advanced cases.^11^

Kato et al^12^ suggested a diagnostic criteria for recognizing patients with severe SB: recent history of tooth noise during sleep, occurring at least 3 to 5 nights a week for a period of 6 months; presence of tooth wear; discomfort or fatigue in the masticatory muscles in the morning; and hypertrophy of the masseter muscle in voluntary clenching. Studies assessing the prevalence of SB in children should adopt patient's complete history and a rigorous physical examination for the diagnosis of SB.

From a scientific point of view, polysomnography is the examination of choice for the diagnosis of sleep bruxism. However, because of its complexity and the need to sleep in a sleep laboratory, polysomnography becomes expensive, thereby hindering its use in clinical practice for many patients, especially children. Thus, alternative diagnostic methods such as BiteStrip^(r)^ used in adults could be developed and validated for children. BiteStrip^(r)^ is used at night to assesses patient's nocturnal activity of masticatory muscles. The method has demonstrated acceptable sensitivity and predictive values ​​as a means of diagnosing SB.^13^

The results of this systematic review revealed different rates of SB prevalence in children in the samples evaluated: 5.9%,^8^ 15.29%,^6^ 35.3%,^7^ 36.8% (pre-school children),^9^ and 49.6% (first graders).^9^ The different rates of SB prevalence in children may be related to several factors. One is the absence of a validated and universal diagnostic criteria for SB in children. Moreover, it appears that studies using questionnaires completed by children's parents as the only resource to assess SB obtained higher SB prevalence rates,^7^^,^ 8 ^,^ 9 while the selected study that combined questionnaires with dental clinical evaluation had the lowest total prevalence.^6^

Prevalence rates show specific diagnostic criteria adopted by the authors. Lam et al^8^ considered as clinically relevant more than three episodes of SB per week represented by the rate of 5.9%. Conversely,the rates by Insana et al^9^ found 36.8% of preschool children and 49.6% of first-grade children with episodes of bruxism at least once a week. However, when assessing 3 to 4 episodes per week, rates decreased to 6.9% and 9.8%, respectively. Serra-Negra et al^7^ reported a prevalence rate of 35.3%. It is important to emphasize that the three studies mentioned above did not perform clinical or polysomnographic assessments for diagnosis of SB; instead, they only used parents' reports. Only one study was conducted with parents' reports, in which case the prevalence was 15.29%. Polysomnography assessment was not used either.^6^

Overall, despite different diagnostic criteria among studies, sex and age differences were observed. Lam et al^8^ found a prevalence of SB of 5.9%, with higher predominance among men (7.7% *versus* 4.7%, OR [95% CI] = 1.69 [1.37 to 2.10], P < 0.001). Prevalence decreased with age for both males and females (linear association P < 0.001). Conversely, Fonseca et al^6^ found that 15.29% (n = 26) were considered bruxists, 15 boys (57.69%) and 11 girls, with no significant correlation between SB and sex (p = 0.595). On the other hand, in the study by Serra-Negra et al,^7^ the prevalence of SB was 35.3%, 56.5% in girls and 43.5% in boys. Insana et al^9^ found that 36.8% of preschool children were reported as bruxists at least one night a week, and 6.7% were reported for more than four nights a week. Conversely, 49.6% of first-graders were reported with SB at least one night per week, and 9.8% were reported for more than four nights a week. Furthermore, girls had a higher rate of no SB in comparison to boys. Thus, three out of four selected studies revealed that SB affected more boys than girls.^6^^,^ 8 ^,^ 9 Additionally, SB decreased with age,^8^ with one study demonstrating an increased prevalence in preschool students in relation to first graders.^9^

It is important to emphasize and try to compare the selected studies within different contexts. One situation refers to where the studies were performed. We found different prevalences in different countries: Brazil (São Paulo^6^ and Minas Gerais^7^), China (Hong Kong)^8^ and USA (Kentucky).^9^

However, what limits and hinders comparison is the criteria adopted for sleep bruxism diagnosis. Were these differences caused by socioeconomic diversity in the different countries and regions assessed or due to lack of diagnostic standardization? Thus, validated, standardized and universal diagnostic criteria are rendered necessary to allow assessment and comparison of the real difference in the prevalence of SB among different countries.

Similarly, comparison of socioeconomic and cultural background between studies using different diagnostic criteria for SB may present conflicting results. How can we compare students from Brazilian public schools with public schools from other parts of the world? How can we compare different age groups if diagnostic criteria are different? Thus, interstudy comparisons are difficult, thereby leaving us with intrastudy comparison only, i.e., the population with which the study was carried out. The study by Serra-Negra et al^7^, who used the Social Vulnerability Index obtained by municipal databases for social classification of families, found that most families were of low social vulnerability (54.2%), while others (45.8%) were of high social vulnerability. Additionally, more than half of children without SB (55.2%) were of low socioeconomic status.

The diagnostic criteria used should also be reflected upon. Only the study by Fonseca et al^6^ conducted clinical assessment based on the American Academy of Sleep Medicine to diagnose SB. Their criteria involved: (1) anterior teeth wear at the incisal border; (2) posterior teeth occlusal wear; (3) parents' report of frequent noises of teeth grinding during sleep; and (4) white line at buccal mucosa and teeth-impressed tongue. Additionally, a questionnaire was given to parents to assess not only the episodes of grinding, but also the child's behavior, the use of medication and duration of breast feeding. Conversely, other studies included parents' report based on different questionnaires,^7^^,^ 8 ^,^ 9 which corroborates differences in prevalence.

The selected studies had methodological limitations. Parents' reports based on questionnaires can be influenced by subjective limitations and memory bias.^8^ On the other hand, clinical assessment is more objective, even though it also has limitations. The method of direct visual observation of dental attrition in the mouth^14^ is another limitation, since it is difficult to ensure whether tooth wear is a result of parafunction or a functional habit, especially in deciduous teeth where occlusal surfaces are physiologically worn.^15^ Despite attrition being regarded as an objective method to record the prevalence of bruxism, it may not indicate the actual level of bruxism. Subjects who were bruxists in the past may have wear facets, even if the habit does not exist anymore; while individuals with recent SB may not show signs of attrition.^16^ Thus, future research may benefit from objective SB measurements and detailed scrutinization of their association with specific health conditions.

Many studies that also showed SB prevalence rates were excluded for assessing not only SB, but the presence of SB associated with oral habits,^17^ TMD,^18^^,^ 19 and occlusal factors.^20^ Excluded studies revealed different SB prevalence rates: 8.4%,^18^ 12.6%,^20^ and 55.3%.^17^ Similarly, studies with the highest SB prevalence were those using questionnaires for SB diagnosis,^17^ in comparison to those combining clinical evaluation and questionnaires.^18^^,^ 20

Sleep bruxism may be associated with other health problems. Therefore, potential factors capable of triggering or perpetuating SB are widely researched in the literature. Thus, altered levels of anxiety and stress, oral habits, malocclusion, hypoventilation, among others, may influence the occurrence of bruxism. It is suggested that a high degree of responsibility and neuroticism, which are individual personality traits, are determining factors for the development of bruxism among children.^21^

Several studies associate emotional disorders - anxiety, depression, aggression, stress - with the bruxism.^21^ A strong correlation was found between bruxism, TMD, high level of anxiety and high-tension personality trait.^22^ One case-control study provided support for the idea that anxiety is a prominent factor for the development of behavioral bruxism in children.^23^ Another study using polysomnography suggests that children with bruxism have a higher degree of excitement, which may be associated with an increased incidence of behavioral and attention problems.^24^

Moreover, it is important to assess the impact of psychiatric disorders on childhood parasomnias,^25^ since individuals affected by Attention Deficit Hyperactivity Disorder (ADHD) treated with medication are more likely to develop bruxism in comparison to individuals affected by pharmacologically untreated ADHD and control.^26^ Conversely, Castelo et al^2^ ^7^ found that children with SB had quality of life scores similar to those without the parafunction.

Occlusal instability during the replacement of deciduous teeth by permanent teeth is another etiological factor that may be related to bruxism in children;^28^ however, another study found no statistically significant relationship between bruxism and occlusion.^20^ Additionally,children with bruxism show greater changes in head positioning in comparison to control groups.^29^ Thus, child's overall health assessment is required in association with dental treatment, thereby performing an integration with Medicine and Psychology in order to yield better treatment results.

Due to the prevalence of sleep bruxism in children, correct and adequate diagnosis is of paramount importance. SB patients should be assisted by specialists in Temporomandibular Disorders and Orofacial Pain, Orthodontics as well as Pediatric Dentistry. Nevertheless, since SB may be associated with psycho-emotional and behavioral disorders, such as anxiety and excitement, a multidisciplinary follow-up is also needed, in which case doctors and psychologists work together to achieve correct diagnosis, recognize perpetuating factors and make the appropriate treatment decision, thus providing children affected by sleep bruxism with quality of life.

## CONCLUSION

A small number of studies met the inclusion criteria of this systematic review. They revealed differences between SB prevalence rates, a fact attributed to lack of standardized and universal diagnostic criteria for SB and subjectivity of some of these criteria. Moreover, some studies were also excluded due to absence of clinical evaluations or total absence of polysomnography assessment for SB diagnosis.

This systematic literature review shows that there is a need for further, evidence-based longitudinal studies with standardized and validated diagnostic criteria including clinical assessment associated with an interview with parents or guardians. Polysomnography should be used as a complementary diagnostic tool in order to obtain more accurate data regarding the prevalence of sleep bruxism in children.
